# Extract of Propolis on Resin-Modified Glass Ionomer Cement: Effect on Mechanical and Antimicrobial Properties and Dentin Bonding Strength

**DOI:** 10.1155/2021/5597837

**Published:** 2021-04-12

**Authors:** Narges Panahandeh, Fatemeh Adinehlou, Seyedeh Mahsa Sheikh-Al-Eslamian, Hassan Torabzadeh

**Affiliations:** ^1^Dental Research Center, Research Institute for Dental Sciences, Shahid Beheshti University of Medical Sciences, Tehran 1983963113, Iran; ^2^Private Practice, Tehran, Iran; ^3^Iranian Center for Endodontic Research, Research Institute of Dental Sciences, Shahid Beheshti University of Medical Sciences, Tehran 1983963113, Iran

## Abstract

This study assessed the effect of addition of aqueous extract of propolis in different concentrations on the mechanical and antimicrobial properties of resin-modified glass ionomer cement (RMGIC). In this in vitro study, powder of Fuji II LC RMGIC was mixed with 25% and 50% aqueous extracts of propolis. Samples (*n* = 15 for shear bond strength, *n* = 5 for flexural strength, and *n* = 20 for the antibacterial activity test) were fabricated using this mixture. The buccal and lingual surfaces of 23 premolars were ground to expose dentin. Tygon tubes were filled with cement, bonded to dentin, and subjected to bond or the flexural strength test in a universal testing machine. Antibacterial activity was assessed using the disc diffusion and well-plate techniques against *S. mutans*. Data were analyzed using one-way ANOVA and Tukey's test. The three groups showed significant differences (*p* < 0.001). The 50% propolis group had the lowest flexural and shear bond strength. The control group had the highest flexural and shear bond strength. No growth inhibition zone was noted around any of the discs. It can be concluded that addition of propolis to RMGIC did not confer any antibacterial activity against *S. mutans* and decreased the flexural and shear bond strength of RMGIC.

## 1. Introduction

Secondary caries refers to development of caries around or beneath the existing restorations over time [1] and is the most important and most common cause of restoration replacement [1–3]. Evidence shows that the type of restorative material significantly affects plaque accumulation and development of secondary caries [4], accumulation of *Streptococcus mutans* (*S. mutans*), and consequently, the risk of development of secondary caries is lower around glass ionomer (GI) restorations compared to composite resin [5]. However, no documented evidence exists regarding the inhibitory effects of GI cements on the occurrence of secondary caries [6]. In other words, GI cements have antibacterial effects on a narrow spectrum of microorganisms and have insignificant bactericidal activity [2]. Thus, researchers added chlorhexidine, antibiotics, and propolis to GI cements to enhance their antibacterial properties [2, 7–11].

GI cements have shortcomings such as moisture sensitivity and low primary strength [12]. However, chemical bonding to enamel and dentin, fluoride release potential, optimal biocompatibility, coefficient of thermal expansion similar to that of tooth structure, and absence of polymerization shrinkage are among the advantages of GI cements [13, 14].

Addition of resin to GI cement increases its bond strength, physical properties, and moisture resistance and improves its esthetic appearance and polishability [15]. Resin-modified glass ionomer cements (RMGICs) are extensively used for patients at high risk of caries and those with multiple carious lesions due to their fluoride release potential and forming an actual chemical and micromechanical bond to tooth structure. They are also used as the restorative material of choice for restoration of cervical lesions [16]. A previous study on patients at high risk of caries due to xerostomia showed a significant reduction in the frequency of caries under RMGIC restorations compared to other restorations [17]. RMGICs have a bond strength similar or higher than that of conventional GI, which may be due to free acid-base reactions in RMGICs, that results in availability of polyacid for longer periods of time and formation of stronger bond.

Researchers are working on addition of chemical agents and plant extracts to dental materials to improve their mechanical and antibacterial properties. de Castilho et al. [18] in 2012 evaluated the effect of addition of chlorhexidine on biological and mechanical properties of RMGIC in vitro (antimicrobial, cytotoxic, and mechanical properties) and in vivo (microbiologic function in affected dentin when used for indirect pulp capping). The results showed that addition of 1.25% chlorhexidine to RMGIC significantly improved its antibacterial properties with no adverse effect on odontoblast-like cells or mechanical properties. Also, the use of mixture of RMGIC and chlorhexidine for indirect pulp capping resulted in complete elimination of *S. mutans* after 3 months.

Propolis is a natural resin produced by the honeybees [19] with antioxidative, antifungal, antiviral, and antibacterial properties [20]. It is used for treatment of candidiasis, acute necrotizing ulcerative gingivitis, gingivitis, periodontitis, and pulpitis in dentistry [3, 21]. There are reports regarding the antibacterial effects of propolis on methicillin-resistant *Staphylococcus aureus* [22, 23]. The bactericidal effects of propolis are attributed to the presence of cinnamic acid and flavonoids in its composition [24]. The ethanolic extract of propolis has been shown to have antifungal effects comparable to those of nystatin [25]. Antimicrobial effects of propolis on anaerobic oral bacteria [26], *Staphylococcus aureus* [27], *Actinobacillus* [28], and oral pathogenic microorganisms such as *Streptococcus salivarius*, *Streptococcus sanguinis*, *Streptococcus mitis*, and *Candida albicans* [29] have been previously documented. Also, a previous study showed that propolis had the greatest effect on *Streptococcus mutans* (*S. mutans*) among Gram-positive and *Shigella* among Gram-negative bacteria [30].

Propolis can have variable properties depending on the geographical region and vegetation of the area from which propolis has been collected as well as the season of collection. Its antimicrobial properties may also vary accordingly [25, 26, 30]. Up to now, about 300 chemical compositions have been identified in propolis, most notably flavonoids, phenols, and aromatic compounds. The ethanolic extract of propolis has been used in most previous studies [2, 7, 31–33]. The effect of addition of ethanolic extract of propolis on the mechanical and antimicrobial properties of conventional GI cement has been previously investigated [32]. GI is a water-based cement, and it is clear that any additive should have been made on a water base composition; thus, this study aimed to assess the effect of addition of aqueous extract of Iranian propolis in different concentrations on the mechanical and antimicrobial properties of RMGIC.

## 2. Materials and Methods

### 2.1. Preparation of Aqueous Extract of Propolis

First, propolis was frozen and micronized using a ball mill. Micronized propolis was immediately added to boiling water in 1 : 2 ratio and stirred for one hour on indirect heat. After two days, the solution was filtered using a filter press. The obtained suspension was centrifuged at 8800 rpm for 30 minutes to obtain the aqueous extract. The obtained extract had a dark brown color. The solution was stored in the dark until the experiment.

The powder to liquid ratio of cement was determined according to the manufacturer's instructions. Mixing was carried out on disposable pads using a spatula. Aqueous extract of propolis [34] in 25% and 50% concentrations [7, 35, 36] was added to the RMGIC. The mixture was used for preparation of samples.

The shear bond strength test was performed on 23 extracted human premolars. The teeth were immersed in 0.5% chloramine T solution for disinfection for 24 hours and stored at 4°C until the experiment. Teeth were then sectioned mesiodistally with a microtome to divide into two halves and eliminate the enamel and expose the underneath dentin. Smooth surfaces were formed on the dentin surface when the specimens were lapped on 600- and 800-grit abrasive paper. Under a stereomicroscope, complete removal of the enamel and the smoothness of dentine surface were confirmed. The specimens were then rinsed off thoroughly with distilled water for at least 30 s.

The study was approved in the Ethics Committee of School of Dentistry, Shahid Beheshti University of Medical Sciences (IR.SBMU.RIDS.Rec.1395.285).

### 2.2. Sample Preparation

Samples were fabricated of Fuji II LC RMGIC in pure form to serve as the control group and mixed with 25% and 50% aqueous extracts of propolis (*n* = 45 for the shear bond strength test, *n* = 15 for the flexural strength test, and *n* = 60 for the antibacterial activity test) (Dr. Jahangir Pharmaceutical and hygienic Co., Lorestan, Iran). For this purpose, RMGIC liquid and powder were mixed on disposable pads using a spatula. Aqueous extract of propolis in 25% and 50% concentrations was added to RMGIC where required.

### 2.3. Microshear Bond Strength Test

Rubber tubes (TBT, Tehran, Iran) with an internal diameter of 2.5 mm and 1 mm length were placed on dentin surfaces and filled with Fuji II LC RMGIC in pure form (control group) and the two mixtures of propolis. Light curing was performed for 20 seconds using a quartz tungsten halogen light curing unit (Turbo Co., Taipei, Taiwan) with a light intensity of 600 mW/cm^2^. The samples were then immersed in distilled water at 37°C for 24 hours. Before shear bond strength testing, cylindrical tubes were cut by a scalpel. The samples were then transferred to a universal testing machine (Zwick Roell, Ulm, Germany). The load at failure was recorded in Newton and converted to megapascal (MPa) by dividing the failure load by the cross-sectional area of each specimen.

### 2.4. Flexural Strength Test

Stainless steel molds measuring 25 × 2 × 2 mm were used for the fabrication of flexural samples. The molds were placed on glass plates and overfilled with RMGIC, and then, another glass plate was used with slight pressure on the mold in order to remove the excess material. Two Mylar strips were used to prevent adherence of samples to the plates. Samples were light cured in five overlapping areas from the core part to the sides. After removal from the mold, the samples were immersed in distilled water at 37°C for 24 hours. They were then transferred to a universal testing machine (Zwick Roell, Ulm, Germany). Load was applied to the sample at a crosshead speed of 1 mm/minute with 0.5 N preload. Maximum load at failure was recorded, and the flexural strength value was determined using the following formula: *σ* = 3Fl/2bh^2^ (*l*, distance between two jigs; *b*, specimen's width; *h*, specimen's height; and *σ*, flexural strength).

### 2.5. Assessment of Antibacterial Activity Using the Agar Diffusion Test

Plexiglass mold was used to fabricate twenty discs measuring 5 mm in diameter and 2 mm in thickness for each group (25% and 50% aqueous extract of propolis and control). *S. mutans* (PTCC 1683) was used for the agar diffusion test. A 5 mm layer of agar was applied in Petri dishes. *S. mutans* suspension was prepared in 0.5 McFarland standard concentration containing 1.5 × 10^8^ bacteria, spread-cultured on blood agar, and incubated at 37°C for 24 hours. Discs were placed on the plates and incubated at 37°C for 48 hours. The diameter of growth inhibition zone around each disc was investigated.

To be assure of the antibacterial activity of propolis in pure form, the well-plate method was used. Blood agar culture medium and *S. mutans* were prepared as above. Using a pipette, two wells were created in agar; 30 *μ*L of propolis was added to one well and 30 *μ*L of ciprofloxacin was added to another well. The plates were then incubated at 37°C for 48 hours, and finally, antibacterial activity was recorded.

### 2.6. Statistical Analysis

Normal distribution of data was assessed using the Shapiro–Wilk test. The data were analyzed using one-way ANOVA. Tukey's test was applied for pairwise comparisons. All statistical analyses were performed using SPSS version 20 software (SPSS Inc., IL, USA) at *p* < 0.05 level of significance.

## 3. Results

The Shapiro–Wilk test showed that data in all three groups were normally distributed (*p* > 0.05). Thus, the three groups were compared using the parametric one-way ANOVA test.

### 3.1. Shear Bond Strength Test Results

Table 1 provides the shear bond strength test results in the three groups. One-way ANOVA showed that the three groups were significantly different (*p* < 0.001). Thus, pairwise comparisons were performed using Tukey's HSD test, which showed that 25% and 50% propolis groups were not significantly different in terms of shear bond strength (*p* = 0.055). The two 25% propolis group and 50% group had significantly lower shear bond strength than the control group (*p* = 0.035 and *p* = 0.001, respectively).

### 3.2. Flexural Strength Test Results

Table 1 provides the flexural strength test results in the three groups. One-way ANOVA showed that the three groups were significantly different (*p* < 0.001). Pairwise comparison of groups using Tukey's HSD test showed that the 50% propolis group had the lowest flexural strength, while the control group had the highest flexural strength; 50% propolis showed significantly lower flexural strength than 25% propolis (*p* = 0.006). The control group exhibited significantly higher flexural strength than 25% and 50% propolis.

### 3.3. Antibacterial Test Results

**Figure 1:**
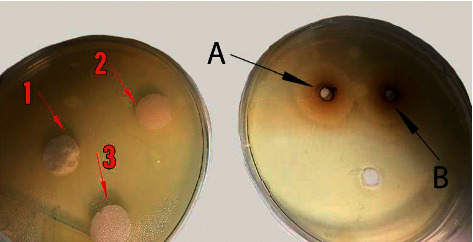
(a) Glass ionomer groups with (1) propolis 0%, (2) propolis 25%, and (3) propolis 50% showing no antibacterial activity against *Streptococcus mutans*. There was no significant difference between the groups (*p* < 0.05). (b) Antibacterial activity of ciprofloxacin (A) and pure propolis (B) against *Streptococcus mutans*. The agar diffusion test showed an inhibition zone of 16 mm for ciprofloxacin and 11 mm for pure propolis.

No growth inhibition zone was noted around the discs. However, pure propolis extract caused a growth inhibition zone with 11 mm diameter. The diameter of growth inhibition zone around ciprofloxacin was 16 mm (Figure 1).

**Figure 2:**
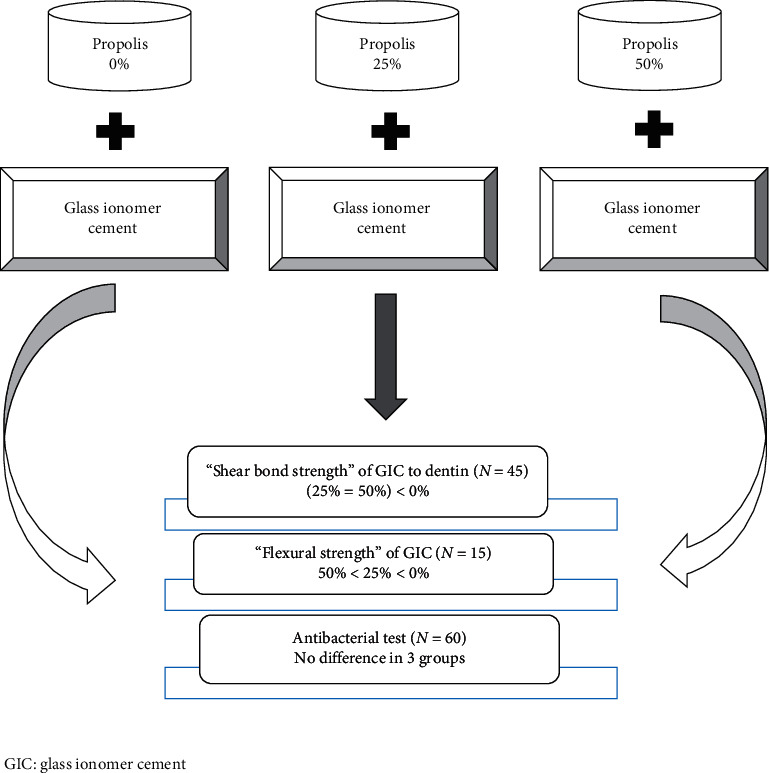
Experimental design and the results of the study.

Figure 2 shows the summery of the overall experimental design, methods used, and results obtained.

## 4. Discussion

At present, the use of extract of the material, instead of its isolated effective components, is preferred since it may have superior therapeutic efficacy due to the synergistic effect of its constituents [2]. The current study evaluated the effect of addition of aqueous extract of propolis in different concentrations on the mechanical and antimicrobial properties of RMGIC.

Knowledge about the mechanical properties of dental materials is important to predict their long-term behavior. In this study, flexural strength and shear bond strength tests were performed to assess the mechanical properties of RMGIC enriched with aqueous extract of propolis. Our results showed that the shear bond strength of 25% and 50% propolis groups was not significantly different; however, the 25% propolis group showed higher shear bond strength, and both values were significantly lower than that of shear bond strength of the control group (*p* < 0.05). The flexural strength test revealed that the 50% propolis group had the lowest and the control group had the highest flexural strength (*p* < 0.05).

It is hypothesized that addition of propolis to RMGIC may increase its antibacterial properties without any deleterious effect on its mechanical features. The results showed that addition of aqueous extract of propolis negatively affected the mechanical properties and did not confer antibacterial activity to the cement (i.e., no growth inhibition zone was observed around the discs made of RMGIC enriched with propolis). However, pure propolis extract created a growth inhibition zone with 11 mm diameter.

Most previous studies on the effect of propolis on antimicrobial activity of GI cements have used ethanolic extract of propolis [2, 31, 33]. However, considering that the GI cements are water based, we assessed the effect of aqueous extract of propolis in this respect.

Some studies have shown that ethanolic extract of propolis increased the bond strength of GI [18, 36]. In contrast, Prabhakar et al. [32] compared the shear bond strength and fluoride release potential of conventional GI cement and GI cement mixed with 1% ethanolic extract of propolis and found no significant difference in shear bond strength of the two groups, while the fluoride release increased significantly. As ethanol may evaporate during setting, the powder: liquid ratio would not be affected by the incorporation of the ethanolic extract. On the other hand, the water in the aqueous extract of propolis will remain in the cement as loosely bond water which may result in decreased mechanical properties.

Evidence shows that propolis contains fatty acids and phenolic compounds [31, 36]. Polyphenols have high molecular weight with unique properties [36]. When propolis is mixed with RMGIC, the hydroxyl phenolic group of propolis reacts with the carboxyl group of RMGIC [32, 36, 37]. In this process, propolis can serve as a space former for different carboxyl groups and create active crosslinks and sodium bridges. As the result of increased crosslinks, the complexity of GI cement increases. However, when aqueous extract of propolis is added to RMGIC, less amount of liquid reacts with powder and crosslinks increase. Since the GI liquid plays an important role in improving the working time and setting time of material, voids may form during mixing of cement with aqueous extract of propolis, which would negatively affect the mechanical properties [2, 36].

The result of this study showed growth inhibition zones with 11 mm and 16 mm diameters around pure propolis extract and ciprofloxacin, respectively. Although chlorhexidine is considered the material to be used as the positive control in the evaluation of antibacterial activity, there have been studies in which ciprofloxacin has been employed for the same purpose, e.g., an investigation by Saputo et al. used ciprofloxacin as a positive control group against *Streptococcus mutans* [38]. The effect of ethanolic extract of propolis on antibacterial properties of conventional GI cement was evaluated, and the results showed that addition of propolis enhanced antimicrobial properties of the cement [2, 7]. Their results were different from the current study, which is probably due to the use of ethanol as a solvent instead of water. Type of solvent of propolis can significantly affect its antibacterial activity [31]. Udoye et al. [31] showed that ethanolic extract of propolis affects a wider spectrum of bacteria, and its bactericidal effect is greater than that of aqueous extract.

Some other factors may also affect the test results. For instance, application of liquid propolis in a well created in the plate caused a growth inhibition zone, while its incorporation into RMGIC and their placement on the agar plate as solid discs did not show any antimicrobial activity. This finding may indicate that release of antibacterial agents from liquids would occur faster and easier than from solid products. Accordingly, Yadiki et al. [37] demonstrated that due to the viscosity of Fuji IX cement and high density of the set cement, release of antibacterial agents from this material would be difficult.

## 5. Conclusion

Addition of propolis to RMGIC did not confer any antibacterial activity against *S. mutans* and in contrast decreased its flexural and shear bond strength.

## Figures and Tables

**Table 1:** Mean ± SD shear bond strength and flexural strength values.

Groups	Shear bond strength (MPa)			Flexural strength (MPa)		
	^∗^Mean ± SD	Minimum	Maximum	^†^Mean ± SD	Minimum	Maximum
Control (RMGI)	2.37 ± 1.29	0.47	5.10	50.26 ± 4.24	45.10	54.16
25% propolis	1.43 ± 1.08	0.20	4.05	34.97 ± 4.49	30.10	41.40
50% propolis	0.55 ± 0.46	0.08	1.80	19.49 ± 8.26	10.91	28.50

^∗^Mean of 15 samples (*n* = 15). ^†^Mean of 5 samples (*n* = 5).

## Data Availability

The data used to support the findings of this study are available from the corresponding author upon request.
